# Elevated fasting glucose is common in lung cancer patients undergoing 18F-FDG PET/CT: A brief report

**DOI:** 10.1016/j.metop.2025.100406

**Published:** 2025-10-14

**Authors:** Bojan Bojanic, Moritz Schwyzer, Thomas Sartoretti, Alessa Fischer, Katharina Binz, Giulia Hofer, Antonio G. Gennari, Matthias Ernst, Martin W. Huellner, Joan Walter, Michael Messerli

**Affiliations:** a Department of Nuclear Medicine, University Hospital Zurich, Zurich, Switzerland; b University of Zurich, Zurich, Switzerland; c Institute of Food, Nutrition and Health, Health Sciences and Technology, ETH Zurich, Zurich, Switzerland; d Curana Endocrinology, Diabetology and Obesity Zurich, Hofwiesenstrasse 350, Zurich, Switzerland; e Department of Endocrinology, Diabetology and Clinical Nutrition, University Hospital Zurich, Zurich, Switzerland

**Keywords:** Lung cancer, Hyperglycemia, Screening

## Abstract

**Objectives:**

To characterize glycemic profiles among patients with lung cancer undergoing standardized capillary fasting blood glucose (cFBG) assessment prior to fluorodeoxyglucose positron emission tomography/computed tomography (FDG PET/CT) imaging.

**Material and methods:**

Consecutive lung cancer patients scheduled for FDG PET/CT were enrolled at the University Hospital Zurich. cFBG was measured before FDG PET/CT and classified according to American Diabetes Association guidelines. Additional analyses were performed in subgroups examined before 11 a.m. and in patients without known diabetes. Multivariable linear regression was used to identify independent factors associated with cFBG levels.

**Results:**

The cohort included 240 lung cancer patients (median age 67 years, IQR 60–73; 41 % female, 99/240; median BMI 24 kg/m^2^, IQR 21–27), of whom 13 % (30/240) had a prior diabetes diagnosis. The median cFBG was 104 mg/dL (IQR 95–115; 5.8 mmol/L, IQR 5.3–6.4). Non-normal cFBG (≥100 mg/dL, ≥5.6 mmol/L) was found in 63 % (151/240) of patients which was consistent in the subgroup presenting before 11 a.m. (58 %, 72/124). Excluding patients with known diabetes, 60 % (125/210) of patients had non-normal cFBG levels, with 8 % (16/210) having levels ≥126 mg/dL (≥7.0 mmol/L), indicative of undiagnosed diabetes mellitus. Multivariable linear regression analysis showed that traditional risk factors such as body mass index were not independently associated with cFBG levels, and neither subcutaneous nor visceral fat were significant predictors.

**Conclusion:**

More than half of lung cancer patients presenting for PET/CT had non-normal fasting glucose levels, even in the absence of traditional risk factors or known diabetes, underscoring the need for improved screening and management strategies in this population.

## Highlights

• More than half (63 %) of lung cancer patients had non-normal fasting glucose levels.
• 60 % of non-diabetic lung cancer patients showed non-normal fasting glucose.
• Traditional risk factors such as BMI, age, and fat distribution were not predictive.
• 8 % of non-diabetic patients had fasting glucose levels indicative of diabetes.

## 1 Introduction

Cancer patients are at increased risk of developing new onset diabetes mellitus (DM) or hyperglycemia irrespective of a preexisting diagnosis of diabetes [1]. Approximately one in five cancer patients is estimated to have coexisting diabetes, a phenomenon likely attributable to shared risk factors [1]. In addition to the well-recognized effects of corticosteroids, various contemporary oncologic therapies can further disrupt glycemic regulation, potentially inducing diabetes in patients with or without pre-existing disease [2]. During chemotherapy, the reported prevalence of hyperglycemia ranges from 10 % to 30 % among patients with malignancies [1]. In the context of lung cancer, deteriorating glycemic control represents a substantial clinical challenge [3]. First, evidence from multiple studies indicates that hyperglycemia, relative to normoglycemia, independently predicts worse overall and disease-free survival, as well as increased recurrence rates in both solid tumors and hematologic malignancies [4]. Specifically for lung cancer, both disease-free and overall survival are adversely affected [4]. Second, although most data are observational, hyperglycemia has been implicated in reducing the efficacy of chemotherapeutic agents [5,6]. Third, suboptimal glycemic control even without formal diabetes diagnosis has been linked to higher rates of complications, such as chemotherapy-induced neutropenia, greater infection risk, and may necessitate premature dose reductions or discontinuation of treatment [1,6]. Fourth, the combined symptom burden of hyperglycemia and cancer therapies can severely impact patients’ quality of life, with those experiencing both cancer and diabetes reporting worse outcomes than those affected by only one of these conditions [7].

Thus, the current study was designed to systematically assess fasting blood glucose status in an unselected series of lung cancer patients presenting for clinically indicated oncologic fluorine-18 fluorodeoxyglucose positron emission tomography/computed tomography (FDG PET/CT) at different stages of care utilizing the standardized fasting blood glucose (FBG) assessment routinely performed at the nuclear medicine department.

## 2 Material and methods

The study was conducted at the University Hospital Zurich, which hosts the region's largest nuclear medicine department [8]. It received approval from the local ethics committee and was carried out in accordance with the Declaration of Helsinki and relevant guidelines and regulations. Written informed consent for the scientific use of medical data was obtained from all patients.

## 3 Study population and questionnaire

This retrospective study included consecutive lung cancer patients (identified by ICD C34 code) undergoing clinically indicated oncologic FDG PET/CT between June 2023 and February 2024. Clinical information was used as recorded in the hospital clinical information system and provided by the patient during examination using a standardized questionnaire.

### 3.1 Capillary fasting blood glucose measurements and definitions

As part of routine clinical practice, patients were instructed to fast before their examination, and patients scheduled in the afternoon were instructed to fast for at least 4 h before the examination. Patients with morning examinations were assumed to be fasting since their last meal the previous day. Capillary FBG was measured after confirming that the patient was fasting for at least 4–6 h by trained nurses using Accu-Chek® Inform II device (Roche Diagnostics, Switzerland). The Accu-Chek® Inform II demonstrated excellent correlation with clinical laboratory glucose measurements (concordance correlation coefficient = 0.95, intraclass correlation coefficient = 0.96), with deviations, when present, being consistently falsely low [[9], [10], [11]]. Depending on the reference method used the average bias of the Accu-Chek® Inform II compared to the respective standard was between -5 and 10 % (i.e. lower values measured by Accu-Chek® Inform II) [10,11].

For this study capillary FBG levels were defined according to the American Diabetes Association: Normal FBG, <100 mg/dL (<5.6 mmol/L); impaired FBG, 100–125 mg/dL (5.6–6.9 mmol/L); and FBG in the diabetes range, ≥126 mg/dL (≥7.0 mmol/L). Additionally, the subgroup of patients with glucose levels ≥200 mg/dL (≥11.1 mmol/L, corresponding to the random glucose threshold for diabetes) was evaluated. Importantly, for the formal diagnosis of diabetes, current guidelines recommend the measurement of fasting plasma glucose using venous plasma samples after at least 8 h of fasting [12]. Visceral and subcutaneous fat were assessed in a standardized, CT-based manner: visceral fat was measured as the distance between the internal surface of the abdominal wall and the anterior aortic wall at the level of the bifurcation, and subcutaneous fat as the distance from the external abdominal wall to the skin at the same level [13]. Known diabetes was defined based on medical records and patient self-report.

### 3.2 Statistical analysis

Comparisons of baseline characteristics were performed using the Mann Whitney-U test, Kruskal-Wallis and Fisher's exact test. Continuous variables are presented as median and respective interquartile range (IQR) and categorical variables are presented as frequencies and respective percentages. Confidence intervals (CI) of proportions are exact binomial 95 % CIs. Spearman's rank correlation was utilized to analyze the relationships between variables. Multivariable linear regression analysis was conducted to identify independent predictors of capillary FBG in multivariate analysis. The analysis was complete-case analysis and performed with log-transformed predictor variables to reduce skewness and mitigate heteroscedasticity. Model adequacy was evaluated through residual diagnostics, assessment of linearity, and checks for multicollinearity. Variables were included in multivariable analysis if their univariate coefficient had a p-value <0.2. With N = 240 and α = 0.05, the study had approximately 80 % power to detect standardized effect sizes (β) of 0.18–0.20 (small to moderate effect) in the multivariable analysis. Three sensitivity analyses were performed: First, patients that had their examination before 11 a.m. in the morning and were fasting for at least 8 h; second, excluding patients with known diabetes; third, the capillary FBG were multiplied a correction factor of 1.08 based on the reported average bias [[9], [10], [11]]. Statistical analyses were performed with R version. All hypothesis testing was two-tailed, and a P-value <0.05 was considered statistically significant.

## 4 Results

A total of 240 consecutive patients with lung cancer presenting for FDG PET/CT, all of whom provided written informed consent, were evaluated in this analysis. The median patient age was 67 years (IQR 60–73), with 41 % (99/240) being female, and 13 % (30/240) carrying a pre-existing diagnosis of diabetes mellitus (Table 1).

**Table 1:** Patient characteristics stratified by capillary fasting glucose levels.

	Overall, N = 240	<100 mg/dL (<5.6 mmol/L), N = 89, 37 %	100–125 mg/dL (5.6–6.9 mmol/L), N = 120, 50 %	≥126 mg/dL (≥7.0 mmol/L), N = 31, 13 %	p-value
**Capillary FBG [IQR],**					<0.001
mg/dL	104 [95, 115]	94 [88, 95]	108 [104, 115]	144 [131, 159]	
mmol/L	5.8 [5.3, 6.4]	5.2 [4.9, 5.3]	6.0 [5.8, 6.4]	8.0 [7.3, 8.8]	
**Female, %**	99 (41 %)	37 (42 %)	52 (43 %)	10 (32 %)	0.534
**Age [IQR], years**	67 [60, 73]	65 [59, 72]	67 [60, 72]	71 [67, 75]	0.028
**BMI [IQR], kg/m2**	24 [21, 27]	24 [21, 27]	23 [21, 26]	27 [23, 29]	0.026
<18	13 (5 %)	4 (5 %)	7 (6 %)	2 (7 %)	
18-25	139 (58 %)	55 (62 %)	72 (60 %)	12 (39 %)	
25-30	58 (24 %)	21 (24 %)	25 (21 %)	12 (39 %)	
30-35	23 (10 %)	7 (8 %)	12 (10 %)	4 (13 %)	
35-40	4 (2 %)	0 (0 %)	4 (3 %)	0 (0 %)	
>40	3 (1 %)	2 (2 %)	0 (0 %)	1 (3 %)	
**Ever smoker, %**	191 (80 %)	67 (75 %)	98 (82 %)	26 (84 %)	0.431
**Known diabetes mellitus, %**	30 (13 %)	4 (5 %)	11 (9 %)	15 (48 %)	<0.001
**Hypertension, %**	90 (38 %)	27 (30 %)	43 (36 %)	20 (65 %)	0.003
**Regular exercise, %**	72 (30 %)	29 (33 %)	37 (31 %)	6 (19 %)	0.369
**Active lung cancer, %**	139 (58 %)	53 (60 %)	68 (57 %)	18 (58 %)	0.916
**Subcutaneous fat [IQR], mm**	20 [14, 27]	19 [13, 24]	21 [14, 27]	20 [15, 28]	0.209
**Visceral fat (IQR), mm**	51 [38, 67]	48 [39, 64]	51 [37, 66]	60 [51, 94]	0.008

BMI – body mass index, FBG – fasting blood glucose, IQR – inter-quartile-range.

**Fig. 1:**
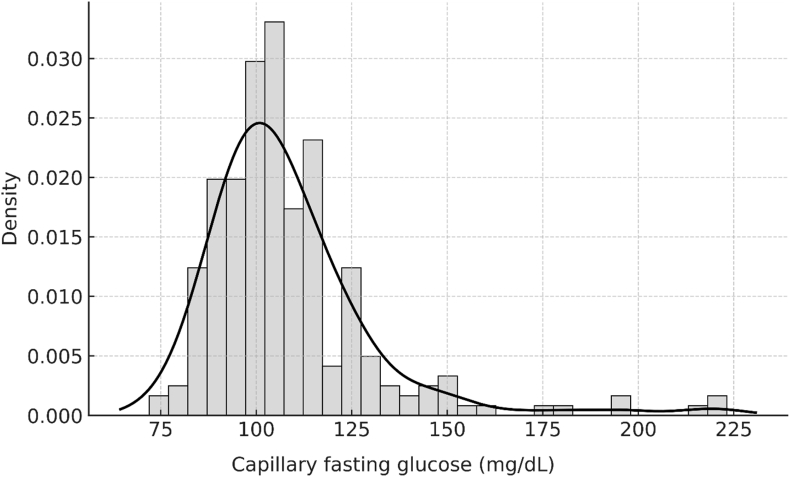
Distribution of capillary fasting glucose (cFBG) Histogram of capillary fasting glucose with overlaid kernel density estimate. The y-axis represents probability density (area under curve = 1).

**Fig. 2:**
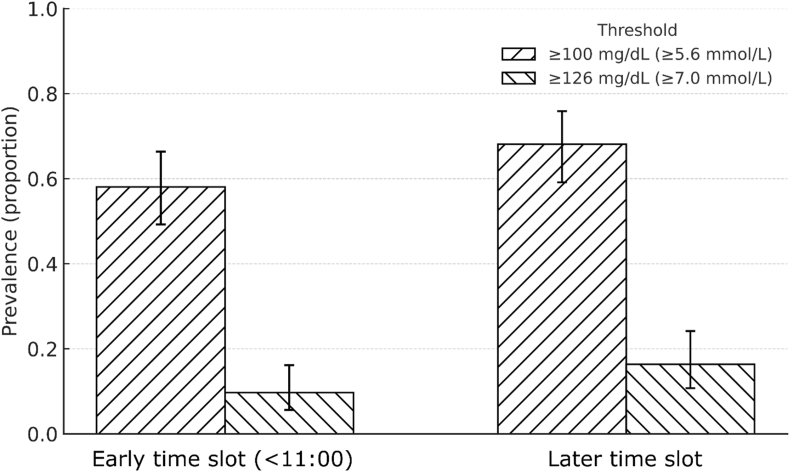
Prevalence of elevated capillary fasting glucose (cFBG) by time of examination. Bars indicate prevalence; error bars represent Wilson 95 % confidence intervals. Patterned fills distinguish thresholds.

Capillary FBG values had a median of 104 mg/dL (IQR 95–115; 5.8 mmol/L, IQR 5.3–6.4). In the cohort overall, 63 % (151/240) exhibited elevated capillary FBG at or above 100 mg/dL (≥5.6 mmol/L), while 13 % (31/240) reached at least 126 mg/dL (≥7.0 mmol/L), and 1.3 % (3/240) registered values at or above 200 mg/dL (≥11.1 mmol/L, Fig. 1). Sensitivity analyses performed for those patients examined prior to 11 a.m. (n = 124) yielded a median capillary FBG of 103 mg/dL (IQR 94–113; 5.7 mmol/L, IQR 5.2–6.3), with 58 % (72/124) recording FBG levels ≥100 mg/dL (≥5.6 mmol/L), 10 % (12/124) at or above 126 mg/dL (≥7.0 mmol/L), and 2 % (2/124) at or above 200 mg/dL (≥11.1 mmol/L, Fig. 2). The difference in FBG between early and later assessments did not reach statistical significance (p = 0.148). Sensitivity analyses were performed with an upward adjustment to account for reported capillary-venous bias [11]. In this setting corrected FBG levels at or above 126 mg/dL (≥7.0 mmol/L were found in 24 % (58/240) of patients in the overall cohort and 19 % (24/124) of patients in the morning subgroup demonstrated.

After excluding the 30 individuals with known diabetes, the median capillary FBG was 103 mg/dL (IQR 95–113; 5.7 mmol/L, IQR 5.3–6.3). Within this subgroup, 60 % (125/210) demonstrated non-normal FBG (≥100 mg/dL; ≥5.6 mmol/L), and 8 % (16/210) had FBG values ≥ 126 mg/dL (≥7.0 mmol/L). After applying an upward adjustment to account for reported capillary–venous bias [11], 19 % (40/240) of patients demonstrated corrected FBG levels at or above 126 mg/dL (≥7.0 mmol/L).

In multivariate linear regression analysis, hypertension (5.8, 95 % CI 1.1 to 10.4, p = 0.015) was identified as an independent predictor of capillary fasting glucose levels. Other variables were not significant, including male sex (p = 0.837), age (p = 0.068), active lung cancer (p = 0.998), smoking history (p = 0.717), regular exercise (p = 0.580), BMI (p = 0.421), subcutaneous fat (p = 0.144), and visceral fat (p = 0.322, Table 2).

**Table 2:** Linear regression analysis for the prediction of capillary fasting glucose.

	Univariate Coefficient (95 % CI), p-value	Multivariate Coefficient (95 % CI), p-value
**Male**	−0.5 (−5.0 to 4.1), 0.837	
**Age, years**	14.3 (−0.5 to 29.2), 0.058	13.7 (−1.0 to 28.5), 0.068
**Active lung cancer**	−0.0 (−4.6 to 4.6), 0.998	
**BMI, kg/m2**	5.0 (−7.2 to 17.1), 0.421	
**Ever smoker**	1.0 (−4.4 to 6.4), 0.717	
**Hypertension**	6.7 (2.1–11.3), 0.004	5.8 (1.1–10.4), **0.015**
**Regular exercise**	−1.4 (−6.2 to 3.5), 0.580	
**Subcutaneous fat, mm**	3.3 (−0.8 to 7.3), 0.111	3.0 (−1.0 to 7.0), 0.144
**Visceral fat, mm**	2.5 (−2.4 to 7.4), 0.322	

BMI – body mass index.

## 5 Discussion

This study assessed the glycemic status in consecutive lung cancer patients who presented for standardized capillary FBG assessment prior to FDG PET/CT examination. We report three major results. First, nearly two-thirds of lung cancer patients presenting for FDG PET/CT demonstrated abnormal capillary fasting glucose values, and this finding remained consistent in sensitivity analyses focused on patients who presented after a longer fasting period in the morning. Second, among patients without a prior diagnosis of diabetes, elevated fasting glucose was detected in 60 %, and 8 % had values at or above 126 mg/dL (≥7.0 mmol/L), corresponding to formal diagnostic thresholds for diabetes mellitus. When applying the upward adjustment to account for reported capillary-venous bias, these findings were even more pronounced with 19 % having corrected values at or above 126 mg/dL (≥7.0 mmol/L). These data corroborate and extend previous findings in melanoma patients [8]. Third, multivariable linear regression analysis showed that BMI, as the main conventional risk factor for elevated blood sugar levels, was not independently associated with these levels. Additionally, none of the traditional risk factors including age, subcutaneous and visceral fat were significant predictors of higher blood sugar levels. This may be explained by the strong impact of cancer-related factors, such as tumor-associated metabolic changes, cachexia, treatment effects, or systemic inflammation, which could overshadow the influence of conventional risk factors [1]. For example, in our cohort most patients were of normal weight (median BMI 24 kg/m^2^), including those with elevated fasting glucose (median BMI 23 kg/m^2^).

These results are consistent with prior reports, highlighting the substantial occurrence of suboptimal glycemic regulation among lung cancer patients in routine clinical care [3]. Existing evidence demonstrates that elevated blood glucose is linked to adverse prognoses in cancer populations, with associations to diminished overall survival and higher rates of disease recurrence across multiple tumor types. In certain malignancies, including lung cancer, poor glycemic control has been shown to negatively affect both overall and disease-free survival [3]. Furthermore, hyperglycemia can undermine the efficacy of chemotherapy, increase the likelihood of adverse events such as neutropenia and infection, and intensify the burdens of cancer treatment, ultimately resulting in worse patient-reported outcomes for individuals contending with both malignancy and diabetes [1,6,7]. Limited real-world evidence suggests that individuals with cancer who develop hyperglycemia are more frequently admitted to the hospital in emergency situations than their normoglycemic counterparts [1]. Timely identification and management of abnormal glucose values may help lower this risk [1]. Moreover, it has been shown that even modest elevations in blood glucose can compete with fluorodeoxyglucose during PET/CT imaging, reducing the contrast between tumor tissue and background and potentially impacting the accuracy of standardized uptake value assessments. Current procedure standards advise fasting for ≥4–6 h before FDG injection and generally proceeding when pre-injection glucose is < 200 mg/dL (<11.1 mmol/L) [14,15]. Elevated glucose levels above this threshold are associated with lower tumor SUVs and markedly reduced true-positive lesion detection in all organs and even earlier in the liver [16]. Whereas diagnostic criteria for diabetes are based on glucose measurements after an 8-h fast (≥126 mg/dL; 7.0 mmol/L), PET/CT protocols typically allow a 4–6 h fast with an upper glucose limit of 200 mg/dL (11.1 mmol/L), a level that already fulfills diagnostic criteria for diabetes in any setting.

Several limitations should be acknowledged when interpreting these results. First, this study's retrospective design and single center setting at a tertiary care hospital limit the ability to draw causal conclusions. Although most potentially eligible patients provided written informed consent, prospective studies are needed to confirm these observations. Second, the analysis relied on capillary glucose measurements. While all tests were performed by trained staff using a calibrated device, capillary values may not fully correspond to venous plasma glucose and are subject to inherent variability. Therefore, an upward adjustment to account for reported capillary-venous bias was performed and confirmed the results. Third, the study reflects real-world fasting conditions, with some individuals fasting less than the recommended 8 h for diagnostic testing in diabetes. To account for this, we performed sensitivity analyses in patients examined in the early morning after overnight fasting. Fourth, factors known to influence glucose metabolism in cancer patients, such as corticosteroid therapy, recent chemotherapy, immunotherapy status, acute stress, or nutritional status, were not specifically examined and may have confounded results. Fifth, visceral and subcutaneous fat distribution was estimated using CT-based linear distance measurements at the level of the aortic bifurcation. While practical, area- or volume-based segmentation provides a more precise assessment of fat distribution.

In conclusion, glucose levels are frequently elevated in lung cancer patients presenting for FDG PET/CT, even in the absence of traditional risk factors or known diabetes. Although imaging quality is often preserved, unrecognized glucose dysregulation may have clinical implications for metabolic health and patient outcomes. Systematic screening and evidence-based strategies for management are needed.

## CRediT authorship contribution statement

**Bojan Bojanic:** Writing – original draft, Project administration, Investigation, Data curation. **Moritz Schwyzer:** Writing – review & editing, Software, Formal analysis, Data curation. **Thomas Sartoretti:** Writing – review & editing, Methodology, Investigation, Formal analysis, Data curation. **Alessa Fischer:** Writing – review & editing, Data curation. **Katharina Binz:** Writing – review & editing, Data curation. **Giulia Hofer:** Writing – review & editing, Data curation. **Antonio G. Gennari:** Writing – review & editing, Methodology, Investigation, Data curation. **Matthias Ernst:** Writing – review & editing, Methodology, Data curation. **Martin W. Huellner:** Writing – review & editing, Project administration, Data curation. **Joan Walter:** Writing – review & editing, Writing – original draft, Supervision, Methodology, Investigation, Funding acquisition, Formal analysis, Data curation, Conceptualization. **Michael Messerli:** Writing – review & editing, Writing – original draft, Supervision, Methodology, Investigation, Funding acquisition, Formal analysis, Data curation, Conceptualization.

## Conflict of interests

Dr. Joan Walter and PD Dr. Michael Messerli are supported by a research grant from the Iten-Kohaut Foundation, Switzerland. Dr. Joan Walter reports research grants from the 10.13039/100002129Swiss Heart Foundation, 10.13039/501100000691the Swiss Academy of Medical Sciences and Bangerter-Rhyner Foundation outside of the submitted work as well as consultant fees of Bayer and 10.13039/100004325AstraZeneca.
